# t(17;19) in Children with Acute Lymphocytic Leukemia: A Report of 3 Cases and a Review of the Literature

**DOI:** 10.1155/2013/563291

**Published:** 2013-01-03

**Authors:** Katherine A. Minson, Pinki Prasad, Susan Vear, Scott Borinstein, Richard Ho, Jennifer Domm, Haydar Frangoul

**Affiliations:** ^1^Department of Pediatrics, Monroe Carell Jr. Children's Hospital at Vanderbilt, Nashville, TN 37232, USA; ^2^Division of Pediatric Hematology/Oncology, Monroe Carell Jr. Children's Hospital at Vanderbilt, Nashville, TN 37232, USA

## Abstract

Several cytogenetic abnormalities identified in patients with childhood acute lymphocytic leukemia (ALL) have been associated with a poor prognosis. There are several case reports in the literature describing t(17;19) in children with ALL. This translocation has been associated with hypercalcemia, coagulopathy, and poor outcome. We present three cases of ALL with t(17;19) treated at our institution and review the outcome of children reported in the medical literature.

## 1. Introduction

Over the past 2 decades, the outcome of children with acute lymphocytic leukemia has exceeded 80% in patients treated in developed countries [1]. Cytogenetic abnormalities, identified in fifty percent of all ALL patients, have long been identified as factors that can influence the risk of relapse. Some of the identified high risk cytogenetic abnormalities include hypodiploidy, t(4;11), and t(9;22)(q34;q11.2) [1, 2]. In current treatment protocols, patients with high risk features are stratified to a more intensive therapy and/or an allogeneic stem cell transplant. 

The chromosomal rearrangement t(17;19), observed in less than 1% of precursor B ALL, has been identified as a poor prognostic indicator. This translocation, which juxtaposes the *E2A *and *HLF* genes, results in the creation of a mutant fusion protein that is thought to play a role in pathogenesis. Furthermore, this translocation results in the disruption of the *E2A* gene which plays an important role in lymphopoiesis [3, 4]. ALL with t(17;19) have been associated with hypercalcemia and coagulopathy, although the mechanism of these findings is unclear [5, 6]. We report on 3 children with t(17;19) ALL and review the outcome of children with this translocation reported in the medical literature.

## 2. Case Report

**Case 1**

A 3-year-old female presented with progressive leg pain. Radiographic evaluation revealed multiple lytic lesions in the lower extremities and pelvis. Laboratory evaluation revealed hemoglobin of 8.6 g/dL, WBC count of 8,400/*μ*L, and platelet count of 162,000/*μ*L. She had hypercalcemia with serum calcium of 16.8 mg/dL (normal 8.5–10.5) and parathyroid hormone-related protein (PTHrP) of 0.62 pmol/L (reference <1.6). Bone marrow evaluation revealed pre-B ALL with t(17;19)(q22;p13) in 15 of 20 metaphases. She received 3-drug induction chemotherapy with dexamethasone (6 mg/m^2^/day for 28 days), vincristine (1.5 mg/m^2^ weekly for 4 weekly doses), and peg asparaginase (2500 IU/m^2^/dose on day 3). She received augmented consolidation therapy with cyclophosphamide (1000 mg/m^2^/dose on day 1), cytarabine (75 mg/m^2^/dose daily for 4 days, days 1–4, and days 8–11), vincristine (1.5 mg/m^2^/dose on days 15 and 22), mercaptopurine (60 mg/m^2^/day days 1–14), and peg asparaginase (2500 IU/m^2^/dose on day 15). However, three months after diagnosis she experienced bone marrow relapse. Re induction therapy was started with doxorubicine (60 mg/m^2^ on day 1), vincristine (1.5 mg/m^2^ weekly for 4 doses), prednisone (60 mg/m^2^/day for 28 days), and peg asparaginase (2500 IU/m^2^/dose weekly for 4 doses staring day 3). Following this salvage therapy bone marrow showed persistent disease. She went to receive dexamethasone (10 mg/m^2^/day, days 1–5), vincristine (1.5 mg/m^2^/dose weekly for 2 doses, days 1 and 8), peg asparaginase (2500 IU/m^2^/dose on day 8), methotrexate (1000 mg/m^2^ infused over 36 hours staring on day 1), thioguanine (100 mg/m^2^/day, days 1–5), and cytarabine (100 mg/m^2^/day for 3 days as continuous infusion days 3–6). Following this therapy, the bone marrow continued to show persistent disease. Final re induction attempt was with clofarabine (52 mg/m^2^/day for 5 days) but she failed to respond with bone marrow evaluation showing 87% blats. The patient died 13 months after her diagnosis with persistent disease.

**Case 2**

A 14-year-old female presented with bone pain, weight loss, bruising, and epistaxis. Her initial blood count demonstrated a platelet count of 35,000/*μ*L, hemoglobin of 5.0 g/dL, and WBC of 13,600/*μ*L with 80% circulating blasts. Other laboratory data included normal calcium and a prolonged INR of 1.9 with low fibrinogen of 76 mg/dL. Cytogenetic evaluation of the bone marrow revealed pre-B ALL with t(17;19)(q22;p13) in all 20 metaphases, with Trisomy 4 and 22. She received 4-drug induction chemotherapy with prednisone (60 mg/m^2^/day for 28 days), daunorubicin (25 mg/m^2^/day weekly for 4 doses starting day 1), vincristine (1.5 mg/m^2^ weekly for 4 weekly doses), and peg asparaginase (2500 IU/m^2^/dose on day 3). Postinduction bone marrow evaluation revealed morphologic remission but 0.4% blasts by flow cytometry. She received augmented consolidation chemotherapy with cyclophosphamide, cytarabine, vincristine, mercaptopurine, and peg asparaginase identical to Case 1. After consolidation she received interim maintenance with high dose methotrexate (5000 mg/m^2^/dose on days 1 and 15). She subsequently underwent an allogeneic bone marrow transplant from an HLA identical sibling. She received a preparative regimen with total body irradiation (1320 cGy divided in 8 fractions over 4 days) followed by cyclophosphamide (60 mg/kg/dose for 2 days). She received a bone marrow graft with a total nucleated cell dose of 3.02 × 10^8^/kg and a total CD34+ cell dose of 5.5 × 10^6^/kg. Graft versus host disease (GVHD) prophylaxis was with cyclosporine and methotrexate (15 mg/m^2^ on day +1 and 10 mg/m^2^ on days +3, +6 and +11). She did not have any GVHD and she was tapered off immune suppression by 5 months after transplant. Eight months after transplant, she experienced bone marrow and extramedullary relapse (skin lesion). She received re-induction chemotherapy with doxorubicine (60 mg/m^2^ on day 1), vincristine (1.5 mg/m^2^ weekly for 4 doses), prednisone (60 mg/m^2^/day for 28 days) and peg asparaginase (2500 iu/m^2^/dose weekly for 4 doses) but she died of disseminated fungal infection 10 months after transplant.

**Case 3**

A 16-year-old male presented with a 2-week history of bone pain and low grade fever. Radiographic evaluation of his lower extremities showed multiple lytic lesions. Laboratory evaluation revealed hemoglobin of 10.4 g/dL, WBC count of 6,000/*μ*L, platelet count of 112,000/*μ*L, calcium of 15.6 mg/dL, PTHrP of <1.1 pmol/L (reference 0–4), and creatinine of 3.75 mg/dL (normal <1.2). Bone marrow evaluation revealed pre-B ALL t(17;19)(q22;p13) in all 20 metaphases. He received 4-drug induction chemotherapy with prednisone, daunorubicin, vincristine, and peg asparaginase. Bone marrow at the end of induction evaluation revealed 1.5% residual leukemia by flow cytometry. He received augmented consolidation chemotherapy (similar to Case 1) but his bone marrow at the end of consolidation therapy demonstrated 1.6% persistent leukemia blasts by flow. He underwent re-induction chemotherapy with cyclophosphamide (400 mg/m^2^/day for 5 days), etoposide (100 mg/m^2^/day for 5 days), and clofarabine (40 mg/m^2^/day for 5 days). Three weeks following this re-induction therapy he died of disseminated fungal infection.

## 3. Discussion

The chromosomal rearrangement t(17:19) in childhood ALL is rare but is increasingly recognized as a clinically significant cytogenetic abnormality. We report three cases to contribute to the growing body of the literature about this translocation and the associated clinical features [4].

Eighteen cases of t(17;19) associated ALL had been previously reported in the English literature (Table 1) [4–14]. There are a number of common clinical features, which are demonstrated in these cases. Some of the first described cases presented with disseminated intravascular coagulation (DIC), a rare finding in childhood ALL [13]. Of the 17 cases with available data, 8 had evidence of DIC either at diagnosis or at relapse.  Case 2 reported here presented with a prolonged PTT and hypofibrinogenemia consistent with DIC.

Another clinical feature commonly seen with t(17;19) ALL is hypercalcemia. This was demonstrated in ten of thirteen previously reported cases with available data. Two of the three cases reported here (Cases 1 and 3) were associated with significant hypercalcemia. It has been suggested based upon previous cases that hypercalcemia in t(17;19) patients is partly parathyroid hormone-related protein (PTHrP) mediated [5]. However, this association was not observed in our patients as both had normal levels of PTHrP.

Perhaps most notably, this translocation has been thought to be a poor prognostic indicator with patients having poor response to initial therapy and early relapse. Of the twenty-one reported cases, all patients have died of progressive disease with the exception of one who was alive on therapy at 1-year after diagnosis with no long-term follow-up data [6]. Two cases presented here were rapidly progressive with poor response to standard therapy leading to early relapse in Case 1 and failure to achieve remission in Case 3.  Case 2 underwent allogeneic bone marrow transplant in first complete remission but still went on to relapse 8 months after transplant. There is one previously reported case of a patient undergoing stem cell transplant in first complete remission with eventual relapse and death [8]. It should be noted that both patients died after relapse and not from complications of transplant. Recently Glover et al. reported on in vitro sensitivity of leukemia blasts from a patient with t(17;19) to dasatinib a tyrosine kinase inhibitor [9]. The patient had a transient clinical response to combination of dasatinib and chemotherapy although he eventually relapsed and died during the re-induction therapy.

The cases reported here are consistent with previous evidence linking t(17;19) ALL with DIC, hypercalcemia, and a poor prognosis. In the current Children's Oncology Group studies, these patients are not classified as very high risk. Based upon previous cases and our experience, we conclude that t(17;19) in ALL should be considered a very high-risk indicator and treated as such. Novel and more aggressive therapies including tyrosine kinase inhibitors, targeted immune therapies, and allogeneic stem cell transplant should be considered in these patients.

## Figures and Tables

**Table 1:** Reported cases of t(17;19) in childhood acute lymphoblastic lymphoma.

Reference	Age (y)/Sex	DIC	Hypercalcemia	Outcome
[13]	16/M	Yes	Yes	Achieved remission, deceased
	15/F	Yes	At relapse	Deceased from relapsed disease
[12]	17/F	NA	NA	Deceased at 2 mo
	17/F	No	No	BMT in 1st CR, deceased from relapsed disease
[8]	11/M	No	Yes	Deceased from relapsed disease
	13/M	No	At relapse	BMT at relapse, remitted then deceased from relapsed disease
[4]	N/A	No	N/A	Deceased from leukemia
	N/A	No	N/A	Deceased from leukemia
[7]	12/F	Yes	NA	Deceased from relapsed disease
	5/F	At relapse	At relapse	Deceased from relapsed disease
[6]	5/F	Yes	No	Deceased from relapsed disease
	14/M	Yes	No	Alive on therapy at 12 m
[11]	14/F	No	At relapse	Deceased from relapsed disease
[14]	14/F	No	Yes	BMT at relapse, deceased from infection after-transplant
	10/M	At relapse	At relapse	Deceased from relapsed disease
[5]	4/M	No	Yes	Deceased from relapsed disease
	12/F	At relapse	At relapse	Deceased from relapsed disease
[9]	10/M	No	No	Deceased from relapsed disease
